# The impact of the COVID-19 pandemic on physical activity and sleep among healthy adults: a systematic review and meta-analysis

**DOI:** 10.3389/fpsyg.2023.1149215

**Published:** 2023-06-29

**Authors:** Dandan Cui, Xiangning Zhang, Jianwei Guo

**Affiliations:** Institute of Artificial Intelligence in Sports, Capital University of Physical Education and Sports, Beijing, China

**Keywords:** COVID-19, lockdown, physical activity, sleep, wearables, elite athletes, healthy adults

## Abstract

**Background:**

The COVID-19 pandemic has had a significant impact on physical and mental health, while physical activity and sleep are two important indicators of the impact that have been explored in recent studies. However, the results of studies with different measurement methods and populations with different levels of physical activity have been diverse in that physical activity and sleep are affected by the COVID-19 pandemic in some studies but not in others. Our study aimed to investigate the impact of the COVID-19 pandemic on physical activity and sleep and the role of measurement methods and populations on results.

**Methods:**

PubMed, Web of Science, and CNKI databases were used to search for related studies systematically. Study characteristics and data on physical activity and sleep were collected and analyzed from each included study. Standardized mean differences (SMDs) with 95% confidence intervals (CIs) were used to estimate pooled effect sizes.

**Results:**

A total of 13 articles were included in the systematic review, 11 of which were included in the meta-analysis. We found that moderate-to-vigorous physical activity (MVPA) time was 0.33 (95% CI 0.07 to 0.59) and sleep quality was 0.37 (95% CI 0.21 to 0.53) decreased, while sleep duration was −0.24 (95% CI −0.28 to −0.20) increased during the lockdown; overall physical activity time had no significant difference (*p* = 0.07) during the lockdown. The “wearables” subgroup had no heterogeneity (*p* = 0.89, *I*^2^ = 0) in sleep duration, while MVPA time measured by subjective scales was not significantly changed. The “elite athletes” subgroup had lower heterogeneity (*p* = 0.69, *I*^2^ = 0) in sleep duration than general adults, while the results of sleep quality for population subgroups were significant and there was no heterogeneity within either.

**Conclusion:**

The COVID-19 pandemic had a significant impact on MVPA time, sleep duration, and sleep quality, instead of overall physical activity time among healthy adults. The results of MVPA time and sleep duration were greatly influenced by the measurement methods, and sleep behavior differed among populations with varying physical activity levels. Thus, when researching physical activity, especially MVPA time, should consider measurement methods, and more attention should be given to differences in populations when researching sleep behavior.

## 1. Introduction

The worldwide COVID-19 pandemic has brought immense distress to all human beings. In the period of early COVID-19, it was estimated that 2.6 billion people (Van Hoof, 2020) were in lockdown or quarantine for an average of 35.38 days in 49 countries (Atalan, 2020). Lockdown slowed down the spread of the coronavirus (Lau et al., 2020), but it influenced public health in both physical health (Werneck and Carvalho, 2020; Amerio et al., 2021; Jurecka et al., 2021; Knowles et al., 2021; Pensgaard et al., 2021; Jia et al., 2022) and mental health (McTiernan et al., 2019; Chandrasekaran and Ganesan, 2021; Khan et al., 2022). The most direct impact of COVID-19 on people was the changes in physical activity, which was reduced due to remote work or study (Bu et al., 2021) or the closure of outdoor activity areas (Ugolini et al., 2021). This had led to changes in life balance, creating difficulties for physical activity. In addition, mental health was affected in ways that cannot be ignored, negative moods (e.g., pressure, irritability, nervous, distress, and worry) were reported in many studies, and corresponding changes in sleep behavior were found in healthy adults (Ingram et al., 2020; Kocevska et al., 2020; Alfonsi et al., 2021; Amerio et al., 2021). Thus, synchronized investigations of physical activity and sleep behavior during the COVID-19 pandemic may provide further evidence for understanding its impact on public health.

The impact of the COVID-19 pandemic on physical activity and sleep behavior among healthy adults has been recently investigated in several studies with inconsistent results. While some studies have reported changes in physical activity (Janssen et al., 2020; Sañudo et al., 2020; Zinner et al., 2020; Buoite Stella et al., 2021; Chouchou et al., 2021; da Silva Santos et al., 2021; Massar et al., 2022) and sleep behavior (Mon-López et al., 2020; Sañudo et al., 2020; Zinner et al., 2020; Lorenzo Calvo et al., 2021; Martínez-de-Quel et al., 2021; Ong et al., 2021) during the lockdown, others have not found any significant changes (da Silva Santos et al., 2021; Vitale et al., 2021). One of the possible reasons could be that the results may be influenced by different measurement methods. Physical activity and sleep behavior can be assessed by subjective scales or wearables. The Pittsburgh sleep quality index (PSQI) (Smyth, 1999) is a traditional subjective way to measure sleep behavior, while the International Physical Activity Questionnaire (IPAQ) (IPAQ Group, 2020) is a common choice to assess physical activity subjectively. Meanwhile, both physical activity and sleep behavior can be measured by wearables (Rosenberger et al., 2016). Given the discrepancy between subjective estimates and objective measures, the results of physical activity and sleep behavior may not be accurately and consistently represented by different measurement methods. Another possible reason could be that different populations have different levels of physical activity in their normal life which is reported to play a moderating role in the impact of the pandemic on one's physical activity and sleep (Martínez-de-Quel et al., 2021). Most typically, elite athletes devote more time to training which results in a much higher level of physical activity than general adults. Furthermore, the inclusion of high-risk populations may confound the study of the relationship between the COVID-19 pandemic, physical activity, and sleep behavior (García-Lara et al., 2022). For instance, healthcare workers were acutely affected by the COVID-19 pandemic, and the disease could affect the physical and mental health of the patients.

In this regard, two existing systematic reviews on physical activity and sleep behavior among healthy adults during COVID-19 suffer from two main limitations: (1) the inclusion of a single population (Jurecka et al., 2021) or confusion of different populations for the study (Hamasaki, 2021) and (2) the inclusion of a single type of measurement method, without comparing both subjective and objective measurement methods (Hamasaki, 2021; Jurecka et al., 2021).

In our study, we conducted a systematic review and meta-analysis of studies examining physical activity and sleep behavior among healthy adults during the COVID-19 lockdown period. We investigated the impact of the COVID-19 pandemic on physical activity and sleep behavior among healthy adults, explored potential differences in these effects across different measurement methods and different populations through subgroup analyses, and, meanwhile, excluded high-risk populations.

## 2. Methods

### 2.1. Search strategy

This systematic review and meta-analysis were conducted according to the Preferred Reporting Items for Systematic Reviews and Meta-Analyses (PRISMA) statement (Moher et al., 2009). The search was performed independently by two authors (XZ and JG) using the databases Web of Science, PubMed, and CNKI. They chose articles that were published after 2020. The search strategy employed Medical Subject Headings (MeSH) terms and free words. The strategies are as follows: (quarantine OR COVID-19 OR lockdown OR coronavirus OR SARS-CoV-2) AND ((physical activity) OR training OR exercise) AND sleep NOT patients^*^ NOT nurses^*^ NOT doctors^*^. Titles and abstracts were screened, and full texts were assessed to ensure that they met the following eligibility criteria.

### 2.2. Eligibility criteria

Eligibility criteria were created based on the PICOs framework: (1) P, Participants: healthy adults who are not healthcare workers and without any risks of disease; (2) I, Intervention: experience the lockdown of the COVID-19 pandemic; (3) C, Comparison: physical activity and sleep behavior data during the lockdown and un-lockdown periods; (4) O, Outcome: the COVID-19 pandemic affects physical activity and sleep or not; and (5) S, Study design: cohort study or cross-sectional study. Any disagreements in the literature screening process were discussed and resolved by consensus between the two authors (XZ and JG).

### 2.3. Data abstraction

The two authors (XZ and JG) extracted the necessary data independently, and all disagreements were resolved through discussion until a consensus was reached. Since the variables that we are concerned with were physical activity and sleep behavior, the necessary data we extracted included overall physical activity time, different levels of physical activity time, sleep duration, and sleep quality, where different levels of physical activity time were not available due to insufficient data on sedentary behavior in the included studies, so we instead performed a detailed analysis of MVPA time. Finally, the necessary data extraction was performed using a Microsoft Excel spreadsheet, and the content of the sheet was author, year, study design, population characteristics, research tools used, date of data collection, main findings, and data regarding physical activity and sleep behavior.

### 2.4. Risk-of-bias assessment

The quality of eligibility studies was assessed using the Joanna Briggs Institute (JBI) critical appraisal tools, including both cross-sectional and cohort (Aromataris and Munn, 2020). The grade standard of risk-of-bias assessment is based on the following criteria: 70% of the answers are “YES” for low risk, 50%−69% of the scores are “YES” for medium risk, and if the score of “YES” is <50%, then it is high risk (TIJ B, 2014). The two authors (XZ and JG) conducted and verified the assessment independently, and all disagreements in the assessment were resolved by an agreement through discussion.

### 2.5. Data analysis

A qualitative analysis was performed to summarize physical activity and sleep behavior among healthy adults during the lockdown period due to COVID-19 compared with a normal period without lockdown.

Quantitative analyses were conducted using Review Manager 5.4, and only SMDs related to statistical models that can be applied to continuous variables were considered. The primary measures were 95% CIs for all the meta-analyses, including overall physical activity time, MVPA time, sleep duration, and sleep quality, respectively. Therein, a fixed-effect model was used for sleep duration, and a random-effect model was used for all other variables.

To assess the stability and reliability of pooled effect size results of the meta-analysis, sensitivity analyses were performed. A leave-one-out sensitivity analysis was used in our study, in which each study was removed from the model once to examine the results. A funnel plot was conducted to detect in meta-analysis where the effect estimates responded to the standard errors.

The difference between studies in a meta-analysis is heterogeneity. To assess heterogeneity in the quantitative analyses, we calculated the *p*-value, with a *p*-value of <0.05 indicating significant heterogeneity (Correll et al., 2018). Additionally, we considered *I*^2^ lower than 25%, between 25 and 75%, and higher than 75% as low, moderate, and high heterogeneity, respectively (Higgins et al., 2003). We pooled the results of the studies with low heterogeneity using fixed-effect models and of the studies with heterogeneity but not considerable heterogeneity using random-effect models. For events with considerable heterogeneity (*p* < 0.05 or *I*^2^ > 50%), subgroup analyses were conducted according to different populations and measurement methods which included a measurement by wearables or not, and whether the population was elite athletes or not for further research.

## 3. Results

### 3.1. Included studies

**Figure 1:**
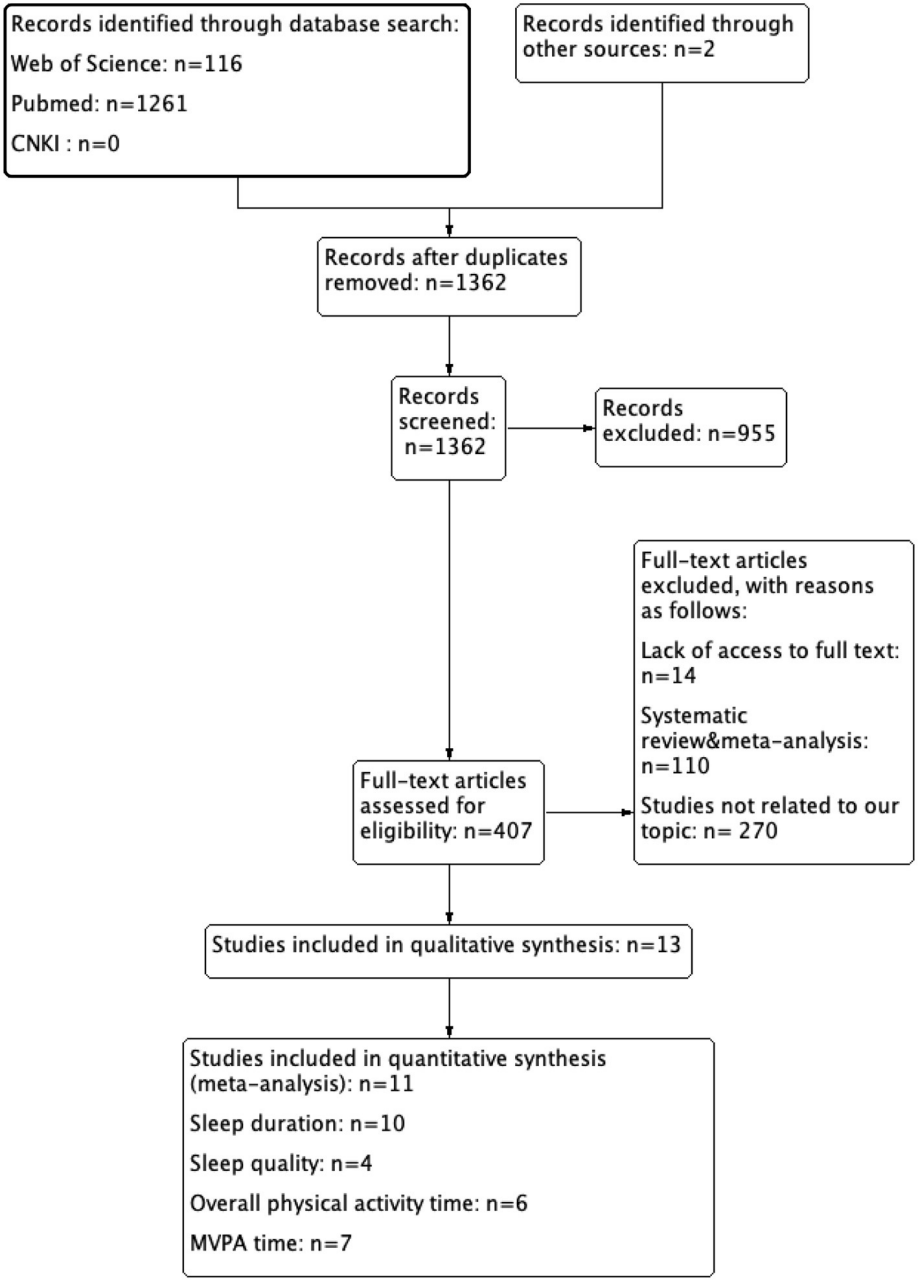
Flow diagram of search strategy results.

A total of 1,379 records were identified by searching PubMed, Web of Science, and CNKI databases, with two additional records from other sources. After the removal of duplicates (17 records) and studies not meeting the inclusion criteria (1,098 records), 407 studies remained. Ultimately, 13 studies were included in the systematic review according to the PICOs criteria, while 11 studies met the inclusion criteria of the meta-analysis. Details of the search results are shown in Figure 1.

### 3.2. Characteristics of included studies

A total of 7,096 participants were included in the studies, except one that did not mention the number of female participants, of which 38.06% were female participants. Of these participants, nine studies included general adults, while five studies included elite athletes in badminton, soccer, basketball, kayaking and canoeing, and track and field. The details are described in Table 1.

**Table 1:** Study characteristics.

**References**	**Study design**	**Population characteristics**					**Methods PA & sleep**	**Date of data collection**	**Main findings**
		***N***	**Female**	**Age**	**Country**	**Population**			
da Silva Santos et al. (2021)	Cohort study	15	9	18.58 ± 2.24	Brazil	Badminton elite athletes	Actigraph GT3X + accelerometers	July, 2019 and July, 2020	Young badminton athletes presented increased sedentary time, and decreased total physical activity time in MVPA, and time in vigorous activities during the COVID-19 pandemic compared to the pre-COVID period, however, there were no significant differences in sleep parameters
Mon-López et al. (2020)	Cross-sectional study	175	25	25.67 ± 5.16	Spain	Soccer elite athletes	PA: Likert scale 1–10 Sleep: Likert scale 1–10	April 12, 2020 to April 19, 2020	The confinement period modified the sleeping behaviourand quality across soccer players
Lorenzo Calvo et al. (2021)	Cohort study	54	20	25.05 ± 6.78	Spain	Basketball elite athletes	PA: Likert scale 1–10 Sleep: Likert scale 1–10	April 16, 2020 to May 5, 2020	The quality of sleep decreased, while sleep hours increased during lockdown
Zinner et al. (2020)	Cross-sectional study	14	8	17.1 ± 1.9	Germany	Kayaker and canoeist elite athletes	Polar M430	March 23, 2020	The German lockdown for containment of the Coronavirus SARS-CoV-2, highly trained kayakers and canoeists spent less overall time training each week with, on average, shorter training sessions and less light-to-moderate physical activity outside of training. They performed more strength training sessions per week and spent longer periods lying down and sleeping during the lockdown
Martínez-de-Quel et al. (2021)	Cohort study	161	60	35.0 ± 11.2	Spain	People older than 18 years old	PA: MLTPAQ Sleep: PSQI	March 16, 2020 and March 31, 2020	A lockdown period due to COVID-19 had a negative impact on the physical activity levels, sleep quality and well-being in a group of physically active Spanish adults, but not in physically inactive participants
Vitale et al. (2021)	Cross-sectional study	89	46	24.7 ± 5.4	Italy	Track and field elite athletes	PA: Subjective Likert scale Sleep: PSQI	May, 2020 to June, 2020	During lockdown, athletes registered delayed bedtime, wake-up time and longer sleep latency during the lockdown compared to pre-lockdown and post-lockdown whereas no changes in total sleep time were reported
Ong et al. (2021)	Cohort study	1,822	941	30.94 ± 4.62	Singapore	Young adults	Fitbit	January 2, 2020 to April 27, 2020	During the lockdown: Time in bed increased by 20 min but without loss of sleep efficiency, PA dropped an average of 42%
Sañudo et al. (2020)	Cohort study	20	9	22.6 ± 3.4	Spain	Young Adults	Xiaomi Mi Band2 wrist-worn accelerometer	February, 2020 March 14, 2020 March 24,2020 to April 3, 2020	During the COVID-19 outbreak, participants spending less time engaging in physical activity, sleeping more hours
Massar et al. (2022)	Cohort study	198	N/A	N/A	Singapore	Young adults	Oura ring	Started at April 15th, 2020 last for 16 weeks	The reopening after lockdown was accompanied by earlier sleep timing, increased physical activity
Buoite Stella et al. (2021)	Cohort study	400	142	35.0 ± 15	Italy	Healthy people older than 18 years old	Smart technology devices	January 2020 March 23, 2020 to March 29, 2020	Daily step count and mean peak heart rate significant reduce suggest the relevant impact of reduced mobility on daily physical activity, although its importance to contrast the virus spread
Ahmad (2022)	Cross-sectional Study	759	573	18–30 51.5% 31–40 18.7% 41–50 14.4% 51–60 15.4%	Malaysia	Adults aged 18–60 years old	PA: IPAQ Sleep: SCI	May, 2020 to September, 2020	The unprecedented COVID-19 outbreak and the lockdown measure during the pandemic have caused significant negative changes in health-related lifestyles and affected the QoL of Malaysian adults
Chouchou et al. (2021)	Cross-sectional Study	400	233	29.8 ± 11.5	Reunion island	Healthy People Older than 18 Years Old	PA: IPAQ Sleep: PSQI	the 35th and 54th days of lockdown	During lockdown, PAs and sleep disturbances happened, which could contribute to an alteration in well-being
Janssen et al. (2020)	Cross-sectional study	3230	2,566	18–24 10.1% 25–34 17.1% 35–49 26.8% 50–64 33.6% 65+ 12.2% Missing 0.3%	Scotland	Healthy people older than 18 years old	PA: IPAQ Sleep: Subjective report	May 20, 2020 to June 12, 2020	From pre-lockdown to lockdown walking decreased, whereas MVPA, sitting and sleep increased, from lockdown to ease levels returned to pre-lockdown for all but MVPA

All the included studies conducted research in both lockdown and un-lockdown contexts, with one study focusing on the lockdown and post-lockdown and the others on the pre-lockdown and lockdown periods. The measurement methods used by the studies are summarized in Table 1. Of the measurement methods, six studies measured physical activity and sleep behavior through the use of wearables such as pedometers or accelerometers (see Table 1), while seven studies measured physical activity and sleep behavior through subjective scales. The results of all the studies were reported in the form of mean ± standard deviation (M ± SD).

### 3.3. Risk of bias within studies

We identified all 13 studies in our research, including seven cross-sectional studies and six cohort studies (Tables 2, 3). Five of the cross-sectional studies (Mon-López et al., 2020; Zinner et al., 2020; Chouchou et al., 2021; Vitale et al., 2021; Ahmad, 2022) were of low-risk, and one study (Janssen et al., 2020) was of medium risk. All six cohort studies (Sañudo et al., 2020; Buoite Stella et al., 2021; da Silva Santos et al., 2021; Lorenzo Calvo et al., 2021; Martínez-de-Quel et al., 2021; Ong et al., 2021; Massar et al., 2022) were of low risk.

**Table 2:** JBI critical appraisal checklist for cohort studies.

**References**	**Were the criteria for inclusion in the sample clearly defined?**	**Were the study subjects and the setting described in detail?**	**Was the exposure measured in a valid and reliable way?**	**Were objective, standard criteria used for measurement of the condition?**	**Were confounding factors identified?**	**Were strategies to deal with confounding factors stated?**	**Were the outcomes measured in a valid and reliable way?**	**Was appropriate statistical analysis used?**	**Johanna Briggs Institute Score**
Ahmad (2022)	Yes	Yes	Yes	Yes	Yes	Yes	Yes	Yes	8
Chouchou et al. (2021)	Yes	Yes	Yes	Yes	Yes	Yes	Yes	Yes	8
Janssen et al. (2020)	No	No	Yes	Yes	Yes	N/A	Yes	Yes	5
Mon-López et al. (2020)	Yes	Yes	Yes	Yes	Yes	N/A	Yes	Yes	7
Vitale et al. (2021)	Yes	Yes	Yes	No	Yes	Yes	Yes	Yes	7
Zinner et al. (2020)	Yes	Yes	Yes	No	Yes	Yes	Yes	Yes	7

**Table 3:** JBI critical appraisal checklist for cross-sectional studies.

**References**	**Were the two groups similar and recruited from the same population?**	**Were the exposures measured similarly to assign people to both exposed and unexposed groups?**	**Was the exposure measured in a valid and reliable way?**	**Were confounding factors identified?**	**Were strategies to deal with confounding factors stated?**	**Were the groups/ participants free of the outcome at the start of the study (or at the moment of exposure)?**	**Were the outcomes measured in a valid and reliable way?**	**Was the follow-up time reported and sufficient to be long enough for outcomes to occur?**	**Was follow-up complete, and if not, were the reasons for loss to follow-up described and explored?**	**Were strategies to address incomplete follow up utilized?**	**Was appropriate statistical analysis used?**	**Johanna Briggs Institute Score**
Buoite Stella et al. (2021)	No	Yes	Yes	Yes	Yes	Yes	Yes	Yes	Yes	N/A	Yes	9
da Silva Santos et al. (2021)	Yes	Yes	Yes	No	Yes	Yes	Yes	Yes	Yes	N/A	Yes	9
Lorenzo Calvo et al. (2021)	No	Yes	Yes	Yes	Yes	Yes	No	Yes	Yes	N/A	Yes	8
Martínez-de-Quel et al. (2021)	Yes	Yes	Yes	Yes	N/A	Yes	No	Yes	Yes	N/A	Yes	8
Massar et al. (2022)	Yes	Yes	Yes	Yes	N/A	Yes	Yes	Yes	Yes	N/A	Yes	9
Ong et al. (2021)	Yes	Yes	Yes	Yes	N/A	Yes	Yes	Yes	Yes	N/A	Yes	9
Sañudo et al. (2020)	Yes	Yes	Yes	No	Yes	Yes	Yes	Yes	Yes	N/A	Yes	9

### 3.4. Qualitative analysis

Changes in physical activity and sleep behavior between the lockdown and the un-lockdown periods in individuals are mixed. For overall physical activity time, six studies were included, and two studies presented a decrease during the lockdown period. Four of seven studies assessed MVPA time and showed decreases, while others showed no significant change. For sleep duration, 10 studies were included, and six studies presented an increase during the lockdown period while others were unchanged. Four studies assessed sleep quality, three of four showed a decrease, and one of four showed unchanged.

### 3.5. Quantitative analyses

For the quantitative analyses, we included 11 studies that assessed physical activity and sleep behavior, while studies (6, 7, 10, and 4) assessed overall physical activity time, MVPA time, sleep duration, and sleep quality, respectively. The exclusion reason for quantitative analyses is that the necessary data were not available in these two studies (Martínez-de-Quel et al., 2021; Vitale et al., 2021). After a leave-one-out sensitivity analysis, the pooled effect sizes of all the quantitative analyses remained stable.

#### 3.5.1. Overall physical activity

**Figure 2:**
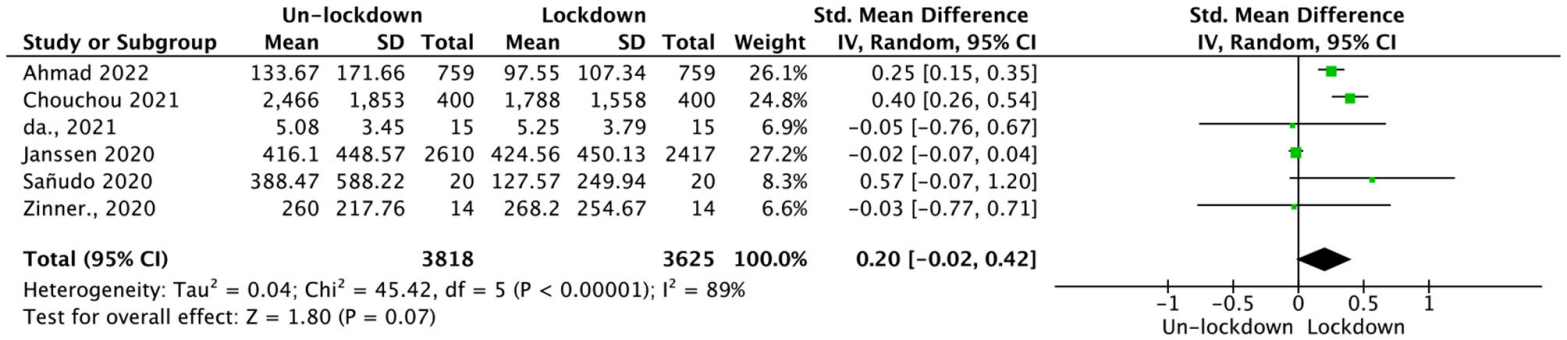
Pooled effect size results of overall physical activity.

Data from six studies including 3,625 participants were analyzed for overall physical activity time. The random-effect pooled Hedges' *g* was 0.20 (95% CI = −0.02, 0.42; see Figure 2), which was not statistically significant (*p* = 0.07), and with high heterogeneity (*p* < 0.01, *I*^2^ = 89%). A subgroup analysis was conducted accordingly.

#### 3.5.2. MVPA time

**Figure 3:**
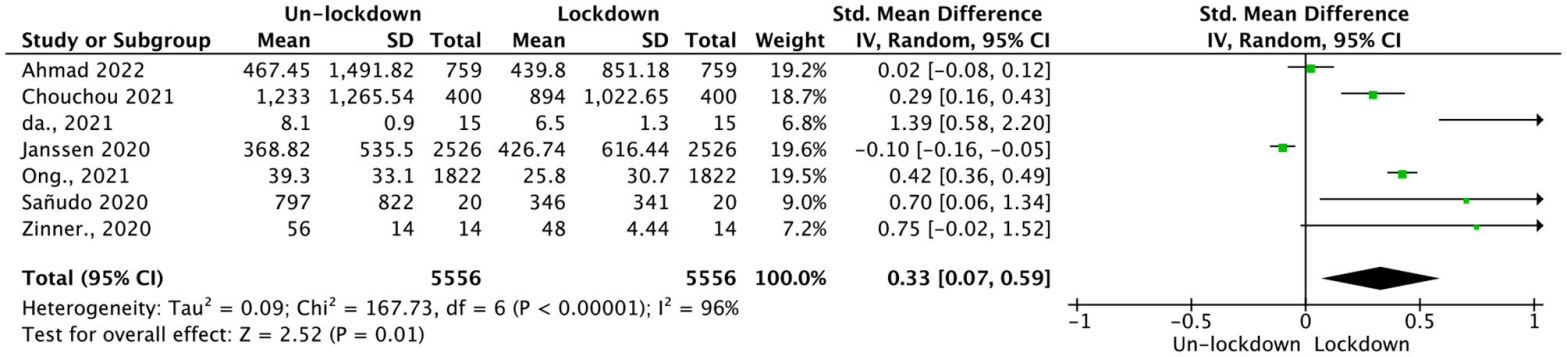
Pooled effect size results of MVPA time.

Data were acquired from seven studies which included 5,556 participants. An analysis of the data revealed a significant association between MVPA time and COVID-19 lockdown, as indicated by Hedges' *g* = 0.33; CI = (0.07, 0.59; see Figure 3). This association was statistically significant (*p* = 0.01), with high heterogeneity (*p* < 0.01, *I*^2^ = 96%). To further explore the source of heterogeneity, subgroup analyses were conducted.

#### 3.5.3. Sleep duration

**Figure 4:**
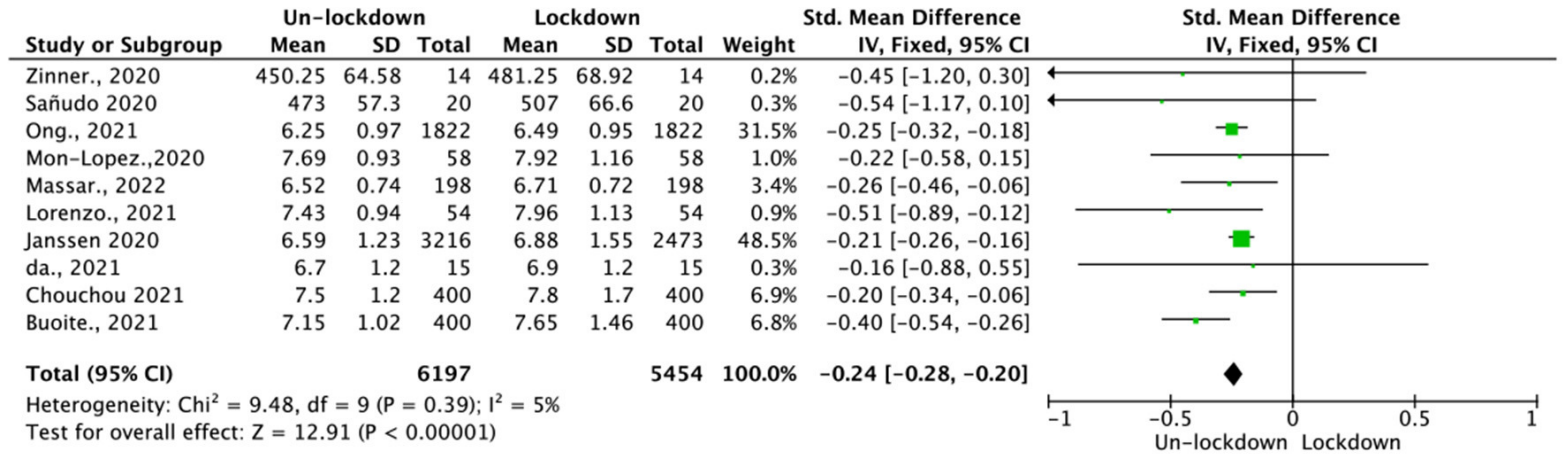
Pooled effect size results of sleep duration.

Data were acquired from 10 studies, which included 5,454 participants. The measurements of sleep duration were based on wearables and subjective scales. Analysis of the sleep duration revealed a low heterogeneity (*p* = 0.39, *I*^2^ = 5%), and consequently, a fixed-effect model was employed. The resulting fixed-effect pooled SMD (Hedges' *g*) was −0.24 (95% CI = −0.28, −0.20), which was statistically significant (*p* < 0.01; see Figure 4).

#### 3.5.4. Sleep quality

**Figure 5:**
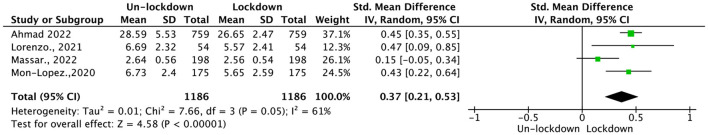
Pooled effect size results of sleep quality.

Data from four studies including 1,186 participants were acquired, and a random-effect pooled SMD (Hedges' *g*) with a 95% CI was conducted. For sleep quality, the results showed a statistically significant Hedges' *g* of 0.37 (95% CI = 0.21, 0.53, *p* < 0.01; see Figure 5). However, moderate heterogeneity was observed (*p* = 0.05, *I*^2^ = 61%); thus, a subgroup analysis was performed.

### 3.6. Subgroup analyses

Given that heterogeneity was observed in the results including overall physical activity time, MVPA time, and sleep quality, subgroup analyses were required. Moreover, we also intended to explore whether there was also an effect of different groupings on the results of sleep duration. Therefore, we conducted related subgroup analyses in measurement methods and populations. After a leave-one-out sensitivity analyses, the pooled effect sizes remained stable.

Six studies were included in the subgroup analysis of overall physical activity time. Neither the population nor the measurement methods showed statistically significant differences before and during the COVID-19 pandemic, with SMDs and 95% CIs for populations of −0.04 (−0.55 to 0.47) and 0.24 (−0.00 to 0.48), respectively, 0.20 (−0.21 to 0.61) and 0.20 (−0.05 to 0.46) for measurement methods, see Table 4.

**Table 4:** Results of subgroup analysis.

**Variables**	**Subgroups**	**Sutdies**	**SMD (95% CI)**	**Overall effect, *p*-value**	**Heterogeniety**	
					*I* ^2^ **, %**	***p*** **-value**
Overall physical activity time	Elite athletes	3	−0.04 (−0.55, 0.47)	0.88	0	0.98
	General adults	4	0.24 (−0.00, 0.48)	0.05	93	< 0.01
	Wearables	3	0.20 (−0.21, 0.61)	0.34	5	0.35
	Subjective scales	3	0.20 (−0.05, 0.46)	0.11	95	<0.01
MVPA time	Elite athletes	2	0.82 (−0.11, 1.75)	0.09	82	0.02
	General adults	5	0.21 (−0.06, 0.49)	0.12	97	<0.01
	Wearables	4	0.69 (0.29, 1.09)	<0.01	56	0.08
	Subjective scales	3	0.06 (−0.14, 0.27)	0.54	93	<0.01
Sleep duration	Elite athletes	4	−0.34 (−0.58, −0.11)	0.004	0	0.69
	General adults	6	−0.24 (−0.28, −0.20)	<0.01	31	0.2
	Wearables	5	−0.25 (−0.33, −0.17)	<0.01	0	0.89
	Subjective scales	5	−0.23 (−0.29, −0.17)	<0.01	51	0.09
Sleep quality	Elite athletes	2	0.44 (0.26, 0.63)	<0.01	0	0.86
	General adults	2	0.43 (0.34, 0.52)	<0.01	0	0.34

In the subgroup analyses of measurement methods in MVPA time, the COVID-19 pandemic had a moderate heterogeneity (*p* = 0.08, *I*^2^ = 56%) effect on MVPA time measured by wearables (*p* < 0.01), while MVPA time measured using subjective scales was unaffected with high heterogeneity (*p* < 0.01, *I*^2^ = 93%). In the subgroups of elite athletes and general adults, there was no association between MVPA time and the COVID-19 pandemic, which is not consistent with the pooled effect result.

Ten studies were involved in sleep duration subgroup analyses. Table 4 shows that both subjective measurement and wearables were significantly associated with lockdown, with SMDs (95% CI) of −0.23 (−0.29 to −0.17) and −0.25 (−0.33 to −0.17), respectively, and no heterogeneity in the results for wearables. Furthermore, both elite athletes and general adults were significantly associated with lockdown state, with SMDs (95% CI) of −0.34 (−0.58 to −0.11) and −0.24 (−0.28 to −0.20), respectively. There was no heterogeneity in the results for elite athletes. Though the sample sizes varied significantly between elite athletes and general adults in the subgroup analysis of sleep duration, the results were robust after sensitivity analysis.

Four studies were involved in the subgroup analysis of sleep quality (see Table 4). The results showed that both elite athletes and general adults were significantly associated with the lockdown, with SMDs (95% CIs) of 0.44 (0.26 to 0.63) and 0.43 (0.34 to 0.52), respectively. There was no heterogeneity detected.

### 3.7. Publication bias

**Figure 6:**
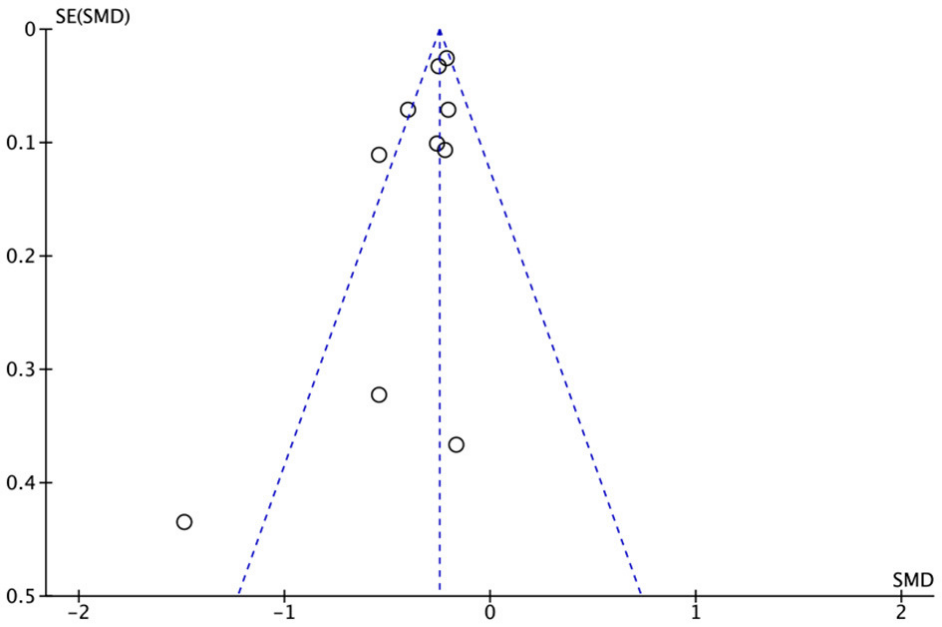
Funnel plot.

We conducted the publication bias assessment using a review manager which is summarized in Figure 6. We used analysis of sleep duration to generate the funnel plot since it included 10 of the 13 studies and covered more than any other analyses. With a visual inspection, there seems to be symmetry which means a low possibility of publication bias (see Figure 6).

## 4. Discussion

### 4.1. Meta-analysis

Our study summarizes the evidence for the prospective association between the COVID-19 pandemic and physical activity and sleep behavior in individuals. Overall, the association of the COVID-19 pandemic with both physical activity and sleep behavior was mixed.

#### 4.1.1. Physical activity

Our results are not consistent with a number of previous studies that have emphasized that there was an increase in overall physical activity time for individuals during the COVID-19 pandemic (Sañudo et al., 2020; Buoite Stella et al., 2021; Chouchou et al., 2021; Massar et al., 2022), but we found no difference. First, the non-significant change may indicate the complexity of the impact of the COVID-19 pandemic on overall physical activity time, and there are different levels of physical activity, which include sedentary, light physical activity, and MVPA. Light physical activity with low exertion may increase during the COVID-19 pandemic, and Wilms et al. (2022) proposed that the lockdown restrictions could cause an increase in low MET activities, such as sedentary behavior (Janssen et al., 2020; Wilms et al., 2022), or increase in housework due to the long period of confinement (Wunsch et al., 2022). Meanwhile, some studies suggested a decrease in MVPA time for individuals during the COVID-19 pandemic (Janssen et al., 2020; Zinner et al., 2020; da Silva Santos et al., 2021), which is consistent with our final findings that MVPA time changed during the COVID-19 pandemic and people spent less time exercising, leading to a decrease in MVPA time, and Ugolini et al. (2021) also suggested that many outdoor leisure activity places were completely closed or restricted, which could reduce people's MVPA time (Karuc et al., 2020). The combination of increased low exertion physical activity and decreased MVPA time led to a non-significant result in overall physical activity time. Furthermore, the decrease in MVPA was more evident in athletes. The main component of physical activity in athletes is training, and some studies (Zinner et al., 2020; da Silva Santos et al., 2021) have shown a decreased training time in athletes during the COVID-19 pandemic which may account for the significant change in MVPA time.

However, there was high heterogeneity in the results for both overall physical activity time and MVPA time, so we further explored the results using subgroup analyses.

#### 4.1.2. Sleep behavior

There is also a growing body of evidence suggesting that the COVID-19 pandemic may have an impact on sleep behavior, but equivocal findings were reported in the previous studies. For example, some studies suggest that sleep behavior (da Silva Santos et al., 2021) such as sleep duration (Vitale et al., 2021) was not affected negatively by the lockdown. However, our meta-analysis reveals that sleep duration increased and sleep quality decreased. Several possible mechanisms explain our results. First, the COVID-19 pandemic may have caused negative moods, many studies have shown that negative moods were related to poor sleep quality (e.g., pressure, irritability, nervous, distress, and worry) (Ingram et al., 2020; Kocevska et al., 2020; Alfonsi et al., 2021; Amerio et al., 2021), and increased negative moods during the COVID-19 pandemic may have worsened people's sleep quality indirectly. Second, less social time could lead to more free time for sleep in individuals. Changes in working or studying status could cause a reduction in socialization (Leone et al., 2020) as people did not need to go outside, and they could manage their own time freely which may be one of the reasons for the increase in sleep duration. Furthermore, the effects of the lockdown on sleep varied across different populations (Kocevska et al., 2020; Alfonsi et al., 2021).

### 4.2. Subgroup analysis

Our results indicated that there was no significant difference in the overall physical activity time in the two subgroups, suggesting that neither of them is the source of heterogeneity in overall physical activity time. Furthermore, we performed subgroup analyses of both measurement methods and populations for MVPA time, sleep duration, and sleep quality.

First, there are discrepancies regarding measurement methods on MVPA time and sleep duration, and the wearables subgroup showed a significant advantage in both the subgroup analysis of MVPA time and sleep duration. One of the possible reasons is that wearables are more accurate and reliable. Studies by Schmidt et al. (2008) and Hagstromer et al. (2010) have shown that objective and subjective measures are independent; in our study, MVPA time measured by wearables showed moderate heterogeneity (*p* = 0.08, *I*^2^ = 56%) and consistent with the results of the pooled effect size, while subjective scales were not. When comparing subjective and objective measurement methods of physical activity (Beagle et al., 2020) and sleep behavior (Grandner and Rosenberger, 2019), wearables were superior to subjective scales. Thus, this may suggest that wearables are more sensitive and may provide more valid results if more studies are conducted using them. Bias in subjective estimates is the other possible reason. In our results, high heterogeneity existed in the factors measured by the subjective scales, suggesting that there were other confounding factors. People may have inaccurate subjective results when they estimate time as they tend to perceive times later than objective measurement results (He et al., 2021), and this possibly confirmed the excessive subjective bias in people's estimates of time which led to the high heterogeneity.

Second, the difference of populations existed in sleep behavior, including sleep duration and sleep quality, of which sleep behavior among general adults was more affected by the pandemic. Physical activity may be the cause of the difference. Our study demonstrated small relative changes in physical activity for the population subgroups. Large differences in absolute values of physical activity between elite athletes and general adults existed, while the former still exhibited higher levels of physical activity than general adults during the lockdown period (Monterrosa Quintero et al., 2022; Shokri et al., 2022). Physical activity has been proven to improve sleep quality and mental health (Werneck and Carvalho, 2020), and some studies suggest that COVID-19 led to physical activity restriction, which may have led to changes in acute endocrine and metabolic profiles (Uchida et al., 2012), and thus inducing the decline in sleep quality. Elite athletes maintained a certain level of physical activity during the COVID-19 pandemic, and they lived a much more regular lifestyle compared to the more unstable lifestyle of the general adults which resulted in the latter being more vulnerable to the COVID-19 pandemic.

### 4.3. Limitations

In this study, we did not include enough studies on healthy adults with different levels of physical activity due to the limited availability of relevant studies, especially healthy adults with high physical activity levels, e.g., elite athletes. The results for both elite athletes and general adults in the MVPA time measure were not significant and inconsistent with the pooled effect size results, which may be due to the small sample size. Although the wearables results were more convincing than the subjective scale, it was not possible to further explore the subjective scale mechanisms and confounders, and it could be the motivation for our research on the improvement of measurement methods.

## 5. Conclusion

We conducted the first systematic review and meta-analysis of physical activity and sleep behavior among healthy adults to assess the impact of the COVID-19 pandemic on both of them during the lockdown period vs. the un-lockdown period. Subgroup analyses were conducted to identify grouping influencing factors. In summary, our results suggest that the COVID-19 pandemic had a significant effect on healthy adults' physical activity and sleep behavior, of which MVPA time and sleep quality decreased, and sleep duration increased. Additionally, measurement methods and populations were found to be valid grouping variables on measures of physical activity and sleep behavior. The results differed between subjective scales and wearables, with wearables being more significantly affected by MVPA time and sleep duration during the COVID-19 pandemic, suggesting that wearables are sensitive and that population subgroups have an impact on sleep duration and sleep quality.

These findings may provide a reference for the preparation of mental health and physical health-related countermeasures under a possible future pandemic. In future studies, the sleep behavior of different populations should be a concern, and we should pay more attention to the accuracy and stability of wearables and subjective scales in measurements for more efficient studies.

## Data availability statement

The original contributions presented in the study are included in the article/supplementary material, further inquiries can be directed to the corresponding author.

## Author contributions

XZ and JG conducted an assessment of included studies, data collection, analysis, and prepared the article for publication after editing and revisions. XZ wrote the initial draft. DC formulated the aims of this study, designed the methodology, made contributions to the revision, and acquired financial support for this study. All authors prepared the article to be published with critical reviews.
